# The novel method to reduce the silica content in lignin recovered from black liquor originating from rice straw

**DOI:** 10.1038/s41598-020-77867-5

**Published:** 2020-12-04

**Authors:** Nghi H. Do, Hieu H. Pham, Tan M. Le, Jeroen Lauwaert, Ludo Diels, An Verberckmoes, Nga H. N. Do, Viet T. Tran, Phung K. Le

**Affiliations:** 1 Institute of Natural Products Chemistry – Vietnam Academy of Science and Technology, 18 Hoang Quoc Viet, Hanoi, Vietnam; 2 Refinery and Petrochemicals Technology Research Center (RPTC), Ho Chi Minh City University of Technology (HCMUT), 268 Ly Thuong Kiet Street, Ho Chi Minh City, Vietnam; 3 Vietnam National University Ho Chi Minh City (VNU-HCM), Linh Trung Ward, Thu Duc District, Ho Chi Minh City, Vietnam; 4 Industrial Catalysis and Adsorption Technology (INCAT), Department of Materials, Textiles and Chemical Engineering (MaTCh), Faculty of Engineering and Architecture, Ghent University, Valentin Vaerwyckweg 1, 9000 Ghent, Belgium; 5 Institute of Environment and Sustainable Development (IMDO), University Antwerp, Groenenborgerlaan 171, 2020 Antwerp, Belgium; 6 Flemish Institute for Technological Research (VITO), Boeretang 200, 2400 Mol, Belgium

**Keywords:** Biochemistry, Biological techniques, Biotechnology, Plant sciences, Environmental sciences, Chemistry, Engineering

## Abstract

Difficulties in the production of lignin from rice straw because of high silica content in the recovered lignin reduce its recovery yield and applications as bio-fuel and aromatic chemicals. Therefore, the objective of this study is to develop a novel method to reduce the silica content in lignin from rice straw more effectively and selectively. The method is established by monitoring the precipitation behavior as well as the chemical structure of precipitate by single-stage acidification at different pH values of black liquor collected from the alkaline treatment of rice straw. The result illustrates the significant influence of pH on the physical and chemical properties of the precipitate and the supernatant. The simple two-step acidification of the black liquor at pilot-scale by sulfuric acid 20w/v% is applied to recover lignin at pH 9 and pH 3 and gives a percentage of silica removal as high as 94.38%. Following the developed process, the high-quality lignin could be produced from abundant rice straw at the industrial-scale.

## Introduction

In recent years, the utilization of lignocellulosic biomass as a renewable source for energy and chemical platforms has been investigated by scientists all over the world^1^. Lignin is one of the most potential renewable and sustainable energy resource which is present in a huge amount of agricultural waste such as maize, rice straw, corn stover, sugarcane bagasse, etc.^2,3^. Amongst them, rice straw accounts for the highest proportion of nearly 50 million tons generated annually in Vietnam, especially in the Mekong delta. However, currently, most of the rice straw is burned resulting in huge emissions of harmful gasses such as NO_x_, CO, CO_2_. Therefore, lignin recovery from rice straw not only prepares a high calorific bio-fuel but also reduces their negative impacts on the environment^4–6^.

The biomass component in rice straw is mainly composed of cellulose, hemicellulose, and lignin which are associated together to form a highly rigid network. Cellulose and hemicellulose are both carbohydrates, while the main building blocks of the lignin structure are phenolic monomers, the so called monolignols^7,8^. These monolignols created the potential of lignin in aromatic compounds production or even biofuel. Additionally, rice straw contains a significant amount of silica, which originates from the soil and enters the roots of the rice plant as mono silicic acid, Si(OH)_4_. Evaporation and transpiration of water in the plant condense the monomeric Si(OH)_4_ species to their saturation point, thus leading to the polymerization into insoluble polysilicon acid^9,10^. Furthermore, the appearance of linkages among components was also confirmed. Lignin associates with polysaccharides, especially hemicellulose, via covalent bonds to form lignin-carbohydrate complexes. Likewise, silica is hypothesized to have interaction with cellulose and lignin^11,12^. The recalcitrant structure of lignocellulosic biomass inhibits bio-refineries such as the fermentation of cellulose for bioethanol, conversion of lignin into value-added chemicals. A lot of silica reduction methods were released but some require harsh conditions or special equipment and others are not efficient^11,13,14^**.** Moreover, there are several desilication methods conducted in mill conditions giving good results, however, this method was not reported about lignin recovery or removing both lignin and silica for gaining cellulose and hemicellulose^9,15,16^.

The pretreatment process is crucial to disrupting the compact structure in biomass, which enhances the yield and success of potential valorization processes. In the case of rice straw, alkaline pretreatment is the outstanding selection for lignin recovery in rice straw since this method is more effective for herbaceous plants than woody plants^17^. During the alkaline pretreatment, lignocellulose is subjected directly to an alkaline solution, which makes the linkages of silica and another component were broken down, and silica was released into an alkaline medium. Moreover, lignin in rice straw and other grass plants possesses a high degree of ester bonds to hemicellulose that is easy to be cleaved by alkaline medium^8,18^. The resulting liquid after separating the pretreated biomass is called “black liquor,” which is originally used to define the waste liquor from the kraft pulping process in paper industries. The alkaline black liquor contains mainly dissolved lignin, silica, and a minor proportion of hemicellulose^19^. That is ideal for effective fractionation of lignin and carbohydrate components as well as silica recovery^8–10^. Among available alkaline agents, sodium hydroxide provides the highest yield of delignification^20^. Moreover, the alkaline pretreatment process is a popular method in the bioethanol production industry, so this method would be suitable for upgrading the bioethanol production technique, while considering about economy, sodium hydroxide is a popular chemical and cheaper than potassium hydroxide or another alkaline. In order to recover lignin and silica from black liquor, Kihlman et al. listed three main methods: acidification, ultrafiltration, and electrolysis^21^. Among these, the usage of acid to precipitate lignin have been dominated the others^22^. Minu et al. have analyzed the effects of mineral acids on lignin from rice straw, while Domínguez-Robles et al. proceeded with these studies on wheat straw^8^. Phosphoric acid offers the highest yield of lignin precipitation but requires a high concentration to reach pH values lower than 4^22^. Hydrochloric acid (HCl) is frequently used in a lab-scale experiment with black liquor obtained from grass plants^23–26^. However, HCl causes corrosion to stainless steel, thus limiting its application in large-scale operation^22^. Under the consideration of economic aspects and feasibility to apply to industrial production, sulfuric is the acid of choice with a reasonable high yield of lignin recovery^27,28^. Likewise, HCl and nitric acid (HNO_3_) had lower efficiency compared to sulfuric acid (H_2_SO_4_)^8^.

The objective of this study is to develop a novel method to reduce the silica content in lignin from rice straw more effectively and selectively. Rice straw was pretreated with an alkaline solution at a mild condition to collect black liquor. The acidification of black liquor from rice straw was studied in a wide range of pH to analyze its precipitation behavior, which has not been reported in any available articles. Precipitates at each pH were analyzed, i.e. their physical properties and chemical structure were analyzed to determine the pH value for the recovery of lignin and silica. The concentration of NaOH was also determined to find the appropriate process for silica reduction and recovery high purity lignin, which can be applied in large scale operation or integration with carbohydrates-oriented processes such as bioethanol production. The characterization of the resulting products was carried out by Fourier-transform infrared spectroscopy (FT-IR), X-ray powder diffraction (XRD), and Thermogravimetric analysis (TGA) to elucidate the chemical structure.

## Materials and methods

### Materials

Rice straw was collected from Cu Chi District, Ho Chi Minh City, Vietnam. The paddy straw was thoroughly rinsed and air-dried under the sunlight until the moisture content of below 15% before being pulverized into pieces of 0.5–2 mm in length and stored in closed bags. The amount of dry matter in rice straw was determined using Sartorius moisture analyzer MA37. The lignin, hemicellulose, and cellulose content of the dry matter were characterized using the method of Nation Renewable Energy Laboratory (NREL) with a report number of TP-510-42618^29^. Sodium hydroxide (NaOH) and sulfuric acid (H_2_SO_4_) in reagent grade were purchased from Merck. All solutions are prepared in distilled water.

### Pretreatment of rice straw

This pretreatment process was carried out at atmospheric pressure. 300 g rice straw was mixed in a 10L boiler with 4.5L NaOH 1w/v%, which was heated to 60 °C in advance. The mixture was simultaneously mixed by agitator (150 rpm) and heated to 90 °C in 15 min and maintained in 2 h at 90 °C. After pretreatment, the mixture was cooled down to 40 °C, followed by vacuum filtration to remove residues. The volume of obtained liquid, i.e., the black liquor, was about 4.3L with a pH value of 12.4. The volume loss of the obtained liquid is mainly due to the efficiency of the process of filtration of rice straws residue to gain black liquor and water evaporation during the mixing step.

### Single-step acidification of the black liquor

The single-stage acidification was conducted to demonstrate the precipitation behavior of the black liquor. Ten samples containing 200 mL black liquor were adjusted with diluted H_2_SO_4_ 20w/v% to reach the pH value ranging from 10 to 1. After the acidification finishes, these samples were left 24 h for aggregation and sedimentation. Each settled mixture was then filtered, and the precipitate was thoroughly washed with deionized water before being dried at 90 °C until a constant mass of solid was obtained. The obtained precipitate was then ground by an agate mortar and pestle. Those treated samples were analyzed to evaluate the effect of pH conditions on the precipitation and select an appropriate condition for two-stage acidification.

### Two-step acidification of the black liquor

The black liquor was acidified to a pH value of 3 by H_2_SO_4_ 20w/v% with two-step adjustments at pH 9 and 3. First, the black liquor had a pH value adjusted to a pH value of 9 and was then left 36 h for silica precipitation. The silica gel was then separated from the liquor by vacuum filtration. Finally, the filtrate was diluted with the low concentrated H_2_SO_4_ 20w/v% to recover lignin at pH 3.

### Elucidation of the effect of sodium hydroxide on lignin obtained from the two-step acidification process

The experiment was conducted to find the appropriate NaOH concentration for the recovery of high purity lignin. The black liquor collected from alkaline pretreatment of rice straw by NaOH with various concentrations (0.5; 1; 2; 4 w/v%) was two-step acidified. The flow chart of the process to recover lignin from rice straw is shown in Supplemental Fig. S1. The purity and recovery yield of the obtained lignin at different experimental conditions was determined. Each experiment was repeated three times and the average value was calculated.

### Analysis methods

The content of ash and non-ash of the obtained precipitations were determined by treating samples at 900 ± 25 °C for 6 h in Nabertherm muffle furnace model LT3/11, therein, the non-ash content was calculated based on the weight difference after calcining. Fourier-transform infrared spectroscopy (FT-IR) spectra of the samples, ranging from 400 to 4000 cm^−1^ with a 4 cm^−1^ resolution, were acquired on KBr pellets using a PerkinElmer Frontier IR instrument. X-ray diffraction (XRD) analysis was performed to demonstrate the structure of samples by using Bruker-D8 Model equipment to record the scattering angle (2θ) and its intensity. Operating conditions were from 10 to 80° (2θ) with a step size of 0.019° and a step time of 43.00 s at ambient condition. A CuKα Ni-filtered radiation (λ = 1.5406 Å) was applied with a working voltage of 40 kV. The TGA results were investigated using Linseis TGA PT 1600. The sample was heated from room temperature to 800 °C with a heating rate of 20 °C/min in argon.

The pH of the black liquors was determined by Thermo Scientific Expert pH meter. The composition of the obtained precipitates, i.e., ash and lignin content was determined by using the NREL/TP-510-42618 method, i.e., by precipitation via two-step hydrolysis using sulfuric acid solution^29^.

The yield of recovered lignin from rice straw was calculated by the equation:

$$\% Yield= \frac{{m}_{rawlignin}\times p}{{m}_{total lignin}}$$

where: ${m}_{rawlignin}$ (g) is the mass of obtained lignin at pH 3. p (%) is the purity of obtained lignin at pH 3. ${m}_{total lignin}$ (g) is the total lignin content in rice straw.

## Results and discussion

### The composition of rice straw

The composition of dry matter in rice straw is determined and the result is shown in Table 1. The rice straw in Vietnam has 50 wt% of cellulose, 22.45 wt% of hemicellulose, and 19.6 wt% of lignin. Meanwhile, the ash percentage of 12.25 wt% represents the silica content in rice straw because the silica content of Vietnam rice straw ash was as high as 80 wt%^30,31^. The pretreatment process successfully dissolves silica and lignin in an alkaline solution with the percentage of lignin and ash (silica) in the black liquor increases up to 51.81% and 25.14%, respectively, thus lignin and silica components can be isolated from the liquor.

**Table 1:** The composition of rice straw and black liquor.

	Cellulose (wt%)	Hemicellulose (wt%)	Lignin (wt%)	Ash (wt%)	Dry matter (wt%)
Rice straw	45.70 ± 0.16	22.45 ± 0.15	19.60 ± 0.18	12.25 ± 0.15	84.73 ± 0.53
Black liquor	11.85 ± 0.37	11.20 ± 0.25	51.81 ± 0.35	25.14 ± 0.22	3.21 ± 0.07

### The precipitation behavior at different pH values

**Figure 1:**
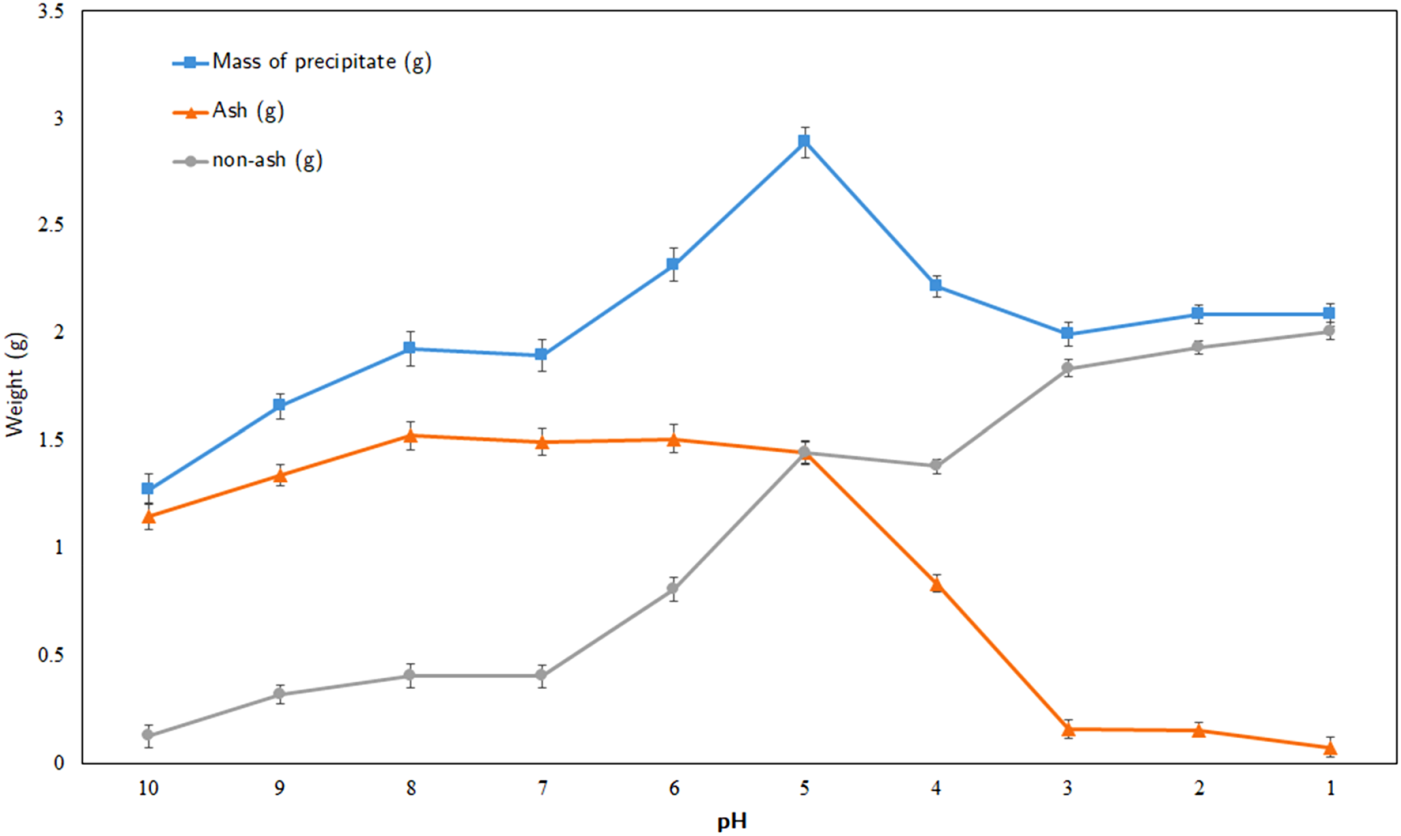
Mass of total precipitate, ash, and non-ash in the black liquors.

The precipitation behavior in acidified black liquors from pH 10 to 1 was described through the physical aspect, weight, and ash (silica) content of the precipitates. The analytical results at each stage of acidification are shown in Fig. 1 indicated the trend of precipitation in black liquor.

**Figure 2:**
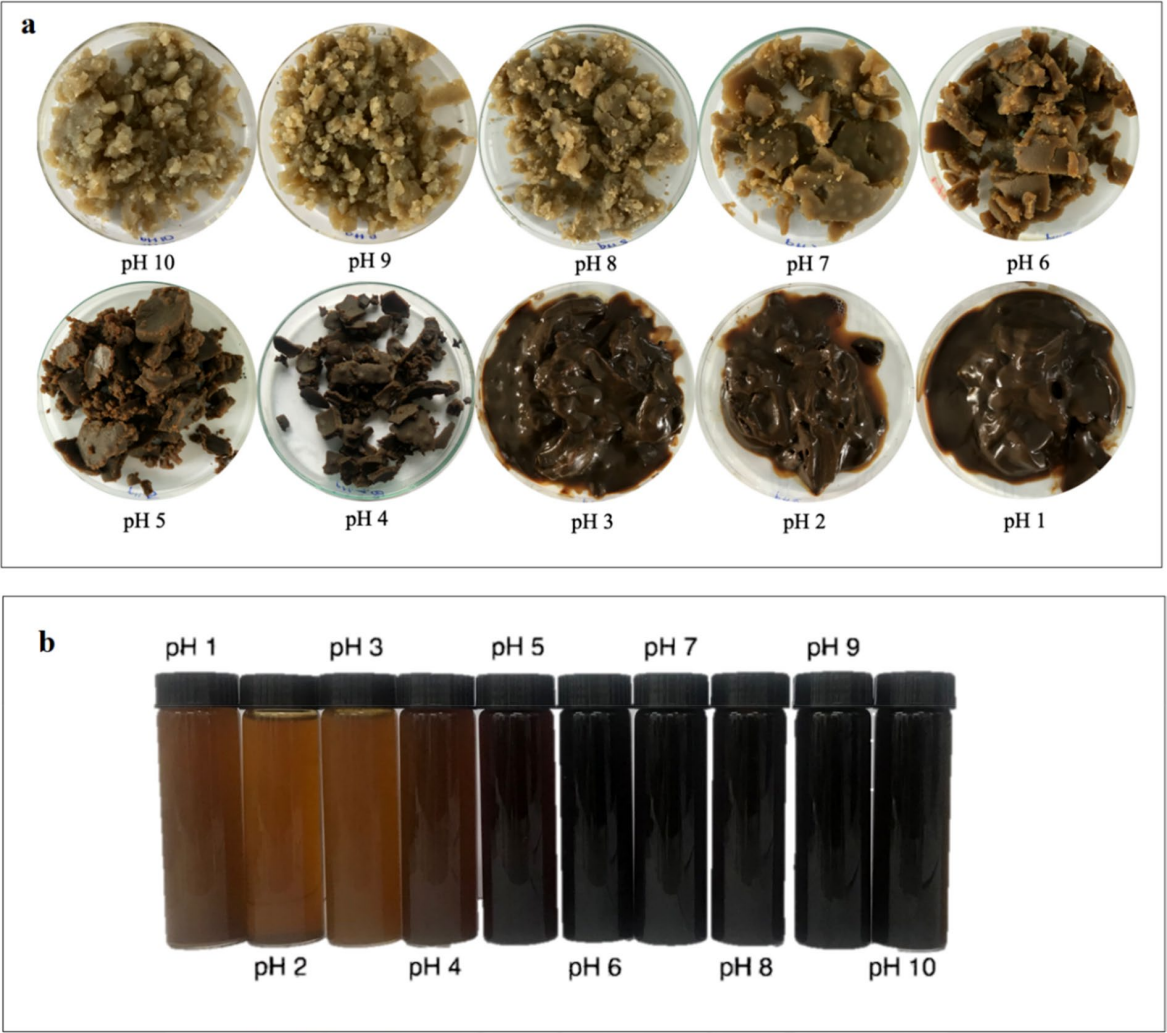
The color variation of the precipitate (**a**) and the filtrates of black liquors (**b**) at different pH values from 1 to 10.

As can be seen, the total mass of the precipitate witnesses a gradual increase with decreasing pH value from 10 to 6 and reaches a peak of 2.89 g at pH 5. When the pH value of the black liquor decreases from 10 to 8, the amount of ash climbs marginally to a peak of approximately 80% at pH 8 and keeps stable until pH 5. According to Aujla et al. and Inglesby et al., silica in rice straw dissolved in the alkaline medium is in the form of sodium silicate at pH 10 and becomes silicic acid when decreasing pH to lower than 10, explaining the gel formation and the appearance of precipitation as seen from Fig. 2a^32^. Zaky et al. proposed two chemical equations to clarify the dependence of silica dissolution Eq. (1) and precipitation Eq. (2) on pH value^33^:

1 $$ {\text{SiO}}_{{2}} + {\text{2NaOH}} \to {\text{Na}}_{{2}} {\text{SiO}}_{{3}} + {\text{H}}_{{2}} {\text{O}} $$

2 $$ {\text{Na}}_{{2}} {\text{SiO}}_{{3}} + {\text{H}}_{{2}} {\text{SO}}_{{4}} \to {\text{SiO}}_{{2}} \cdot {\text{H}}_{{2}} {\text{O}} + {\text{Na}}_{{2}} {\text{SO}}_{{4}} $$

Therefore, the presence of dissolved silica (as silicic acid) in black liquor and the formation of sodium silicate precipitation upon acidification is the main reason for the rising and stability of ash content. The precipitate due to gel formation can be ascribed to the formation of the silicic acid hydrate. In the range of pH 5–7, the non-ash content witnesses a significant increase from 20 to 63%. However, the ash content plummets between pH 4 and 3 from 35% to 16 which is fit to the considerable drop of total precipitate weight. According to Minu et al. the acidification of black liquor to pH lower than 4 leads to the re-dissolution of silica^8^. Hence, at pH 3 and lower, the weight of precipitate remains unchanged due to lignin content. Thus, lignin can be recovered at pH 3 or lower.

The appearance and color change of the precipitate is shown in Fig. 2a. Black liquors treated in a pH range from 10 to 8 are in a state of a dense gel, which could be easily separated by filtration. Substantial precipitation takes place when the pH of black liquor reaches a value of 10 due to the presence of silicates^27^. The color of the precipitate at pH 10 to 8 is light golden brown while at pH 7 and lower, it noticeably changes to dark brown, which is proportional to the decolorization of the black liquors^32^. The appearance of brown shade in the precipitates is evident from the presence of lignin chromophore^34^. Therefore, the co-precipitation of lignin and silica occurs at pH 7 and lower. The sedimentation of treated liquors occurs instantaneously after the pH reached 3 or lower values. The brown sediments settle to the bottom of the liquid phase, which takes at least 5 h to accomplish and the obtained precipitate at this pH is in a slurry state. This can be explained by the precipitation of lignin at low pH when lignin acts as a hydrocolloid due to the impacts of protonation of acid groups in lignin structure^26^.

The color alteration of processed liquors at different pH values is illustrated in Fig. 2b. The filtrates are remaining dark brown until pH down to 5 before change into opaque reddish-brown. Mussatto et al. reported the color change of the black liquor which originated from brewer’s spent grain was observed from pH 12 to 2^34^. Garcia et al. demonstrated the transformation of black liquor from the treatment of *Miscanthus Sinensis* with decreasing pH from 12 to 1^27^. Filtered liquors in both studies, which were collected from the single-step precipitation, turned from dark brown to light brown. Alkaline-soluble derivatives, which are generated during lignin degradation such as quinones, carbonyl groups, carboxylic acids, hydroperoxyl radicals, phenolic hydroxyl groups, are responsible for the dark color of the black liquor^35^.

### Clarification of chemical structure

#### FT-IR analysis

**Figure 3:**
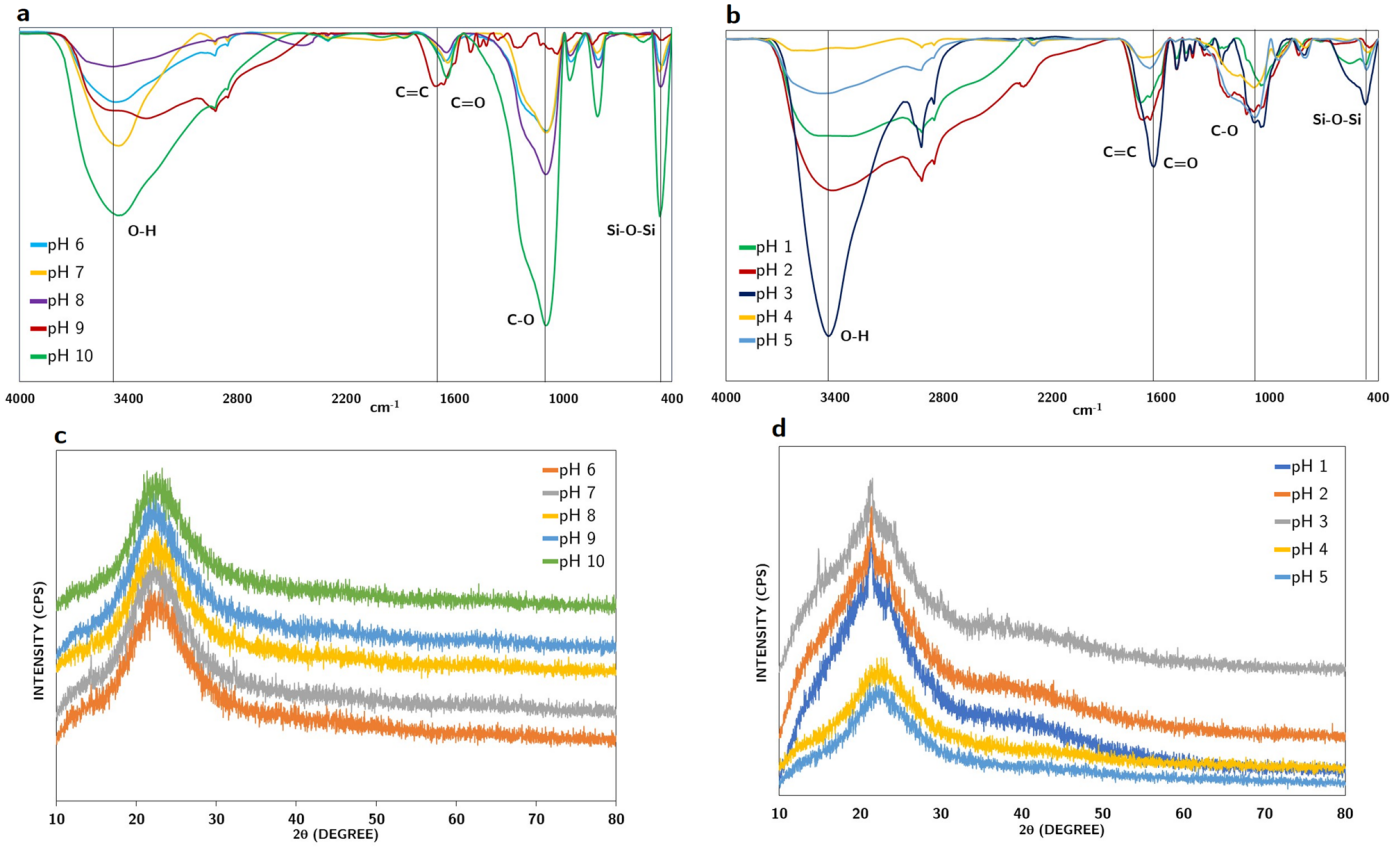
FTIR and XRD spectrums of the precipitate from pH 10 to pH 6 (**a**,**c**) and from pH 5 to pH 1 (**b**,**d**).

The precipitates from pH 10 to 1 were analyzed using FTIR in the 4000–400 cm^−1^ region and shown in Fig. 3a,b. Based on the FTIR spectroscopy band assignments of the sample in Table S2, the spectra of all precipitates obtained at pH values ranging from 10 to 1 exhibit most of the lignin and silica bands (Table S2). In particular, the intensive bands between 3000 and 3500 cm^−1^ are assigned to OH stretching vibrations. The lignin bands are present around 1510 cm^−1^ and 1605 cm^−1^ for aromatic skeletal vibration (C=C) of lignin (guaiacyl or syringyl) whilst the absorption bands around 1604 cm^−1^ and 1735 cm^−1^ can be assigned to the C=O stretching of lignin^36^. The aromatic ring group is also found in the region of 800 cm^−1^ and 833 cm^−1^^37^. The presence of silica is indicated by the Si–O–Si bending region (458–561 cm^−1^) and the bands from 950–1000 cm^−1^ of Si–O–Si asymmetric stretching^38–40^.

Figure 3a,b also illustrates that except for the domination of the absorption bands from 3000–3500 cm^−1^, the spectra of the precipitates from pH 10 to 8 are significantly affected by silica bands. However, the peak of silica decreases from pH 7 steadily (Fig. 3a) and from pH 3 to 1 (Fig. 3b), the effect of silica bands is negligible, whereas the lignin bands are more pronounced in the spectra of precipitates from pH 3–1, which also supports the assumption about the recovery of lignin at pH 3 with less impurity.

#### XRD analysis

Figure 3c, 3d show an X-ray powder diffraction pattern of the precipitate from black liquors at several pH values^41^. In general, the absence of peaks of likely impurities such as sodium sulfate and other salts or metals confirms the purity of recovered products^42,43^. The peak between 17° and 30° recorded in the precipitate from pH 10 to pH 6 indicates amorphous silica according to Liu et al. and Tinio et al.^44,45^. Together with the featureless diffractograms, the appearance of a diffuse maximum at 22.5° indicates the amorphous nature of silica existing in the recovered precipitates in this range of pH^46,47^.

The width of the peak of products at pH 5 and pH 4 is broader from precipitates obtained at higher pH values indicating a higher amorphous degree (Fig. 3c,d). This might be relevant to the strong alternation in the ash and organic contents in the precipitate at two pH levels. The similar XRD patterns of products precipitating at pH lower than 3 are illustrated by a broadening of the peaks between 10° and 45° (2θ), which indicates the domination of amorphous structure in precipitates at low pH. Therefore, XRD diffractograms in this study prove the correlation in the distribution of silica and organic compounds in recovered products. Kauldhar et al. claimed that the XRD pattern of standard pure lignin showed a major diffraction peak between 23° and 32° (2θ)^10^.

#### TG analysis

**Figure 4:**
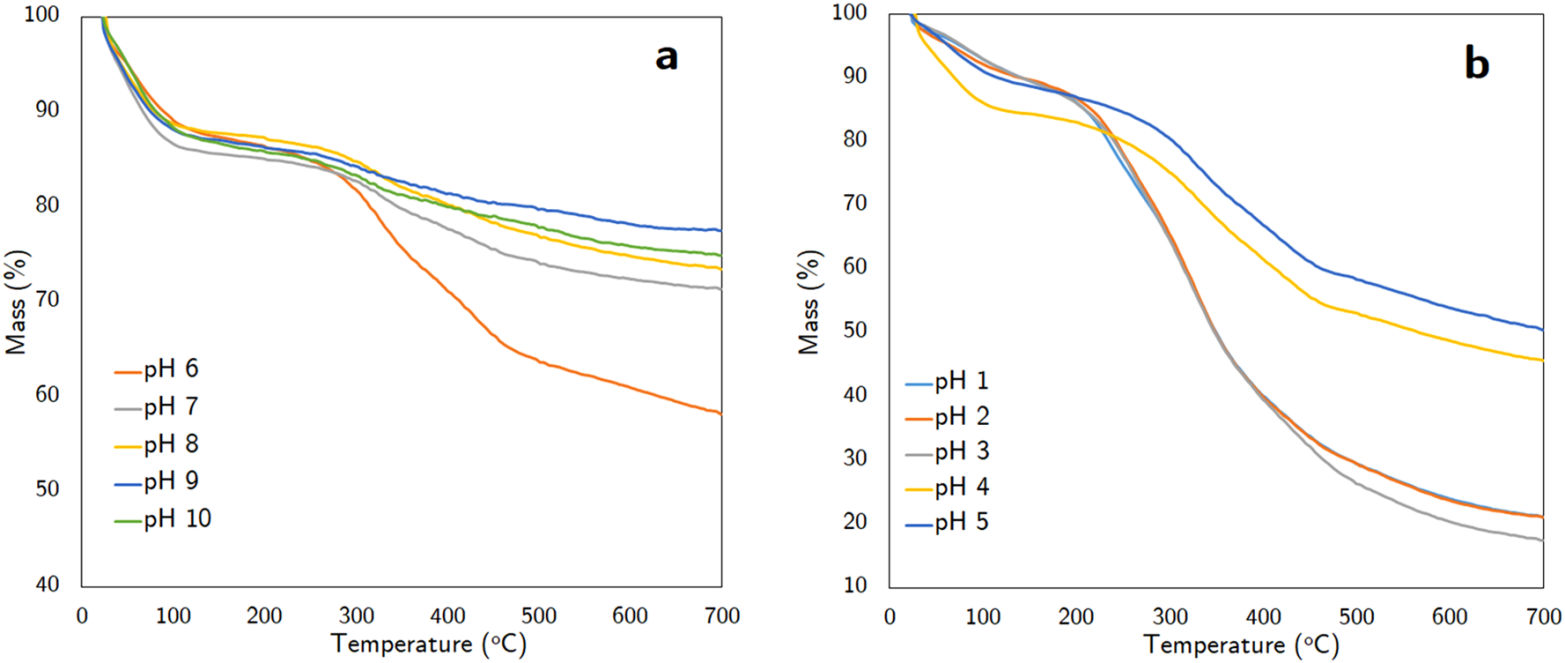
TG analysis of the precipitates from pH 10 to pH 5 (**a**) and from pH 4 to pH 1 (**b**).

The thermogram of recovered products precipitated at basic pH from 10 to 1 is shown in Fig. 4. We obtained four temperature zones, as interpreted in the following. The precipitated products at pH 10, pH 9, pH 8, and pH 7 witnessed a thermal degradation by about 25 wt%. The second group including profiles of pH 6, pH 5, and pH 4, had the total mass loss ranging from 41–53%. The last group, which contains TGA curves of precipitate obtaining at pH 3, pH 2, and pH 1, experienced about 80 wt% loss due to thermal degradation.

The first stage occurred below 100 °C due to the evaporation of physically adsorbed water. The thermogram of precipitates at pH 10 to pH 4 reported the fluctuated mass reduction in the range of 10–14 wt%, while the weight loss of products precipitated at pH 3 and lower decreased to 7%. The adsorption of water on the surface of OH groups of silica is responsible for the higher moisture content of recovered products at pH 10–4^10^.

The second stage was observed around 100 °C–260 °C, which indicated the decomposition of polysaccharides, aliphatic alcohols, and acids ^48^. At pH ranging from 10 to 4, the mass loss was less than 6 wt%, which confirms the negligible presence of non-lignin compounds co-precipitating with silica, whereas 10–14% of weight loss was reported from samples recovered at pH 3 to pH 1. The noticeable loss implies the coprecipitation of lignin and hemicellulose, which is evident in the existence of lignin-carbohydrates complexes ^49^. Also, organic compounds were likely converted into intermediates, which did not affect the weight reduction^42^.

The increase in thermal degradation occurred mainly in the temperature interval of 260 °C–480 °C. From pH 10 to pH 7, the mass loss is approaching 8 wt%, then jumps to the range of 18–24% at pH 6 to pH 4. The weight loss reached 50 wt% at pH 3 and remained the same at pH 2 and pH 1. This third stage included the subsequent transformation of intermediates, which were formed in the second stage, into gaseous components and tar ^42^. The decomposition of lignin happened strongly in this stage. Since lignin consists of aromatic building blocks with diverse branching, the thermal properties of lignin are varied due to the difference in biomass origin^10^.

At the fourth stage over 480 °C, a negligible mass loss of less than 5% was recorded from thermograms of samples from pH 10 down to pH 6. The weight loss of samples at pH 5 and pH 4 were 9%. At pH 3 and lower, the thermal degradation of samples rose to 15 wt%.

In general, the results of all experiments, including FTIR, XRD analysis, and TG analysis, indicated that the lignin and silica precipitates were able to be recovered at acidic pH value (pH 1–3) and basic pH value (pH 8–10), respectively.

#### Determination of pH for removing silica content

**Figure 5:**
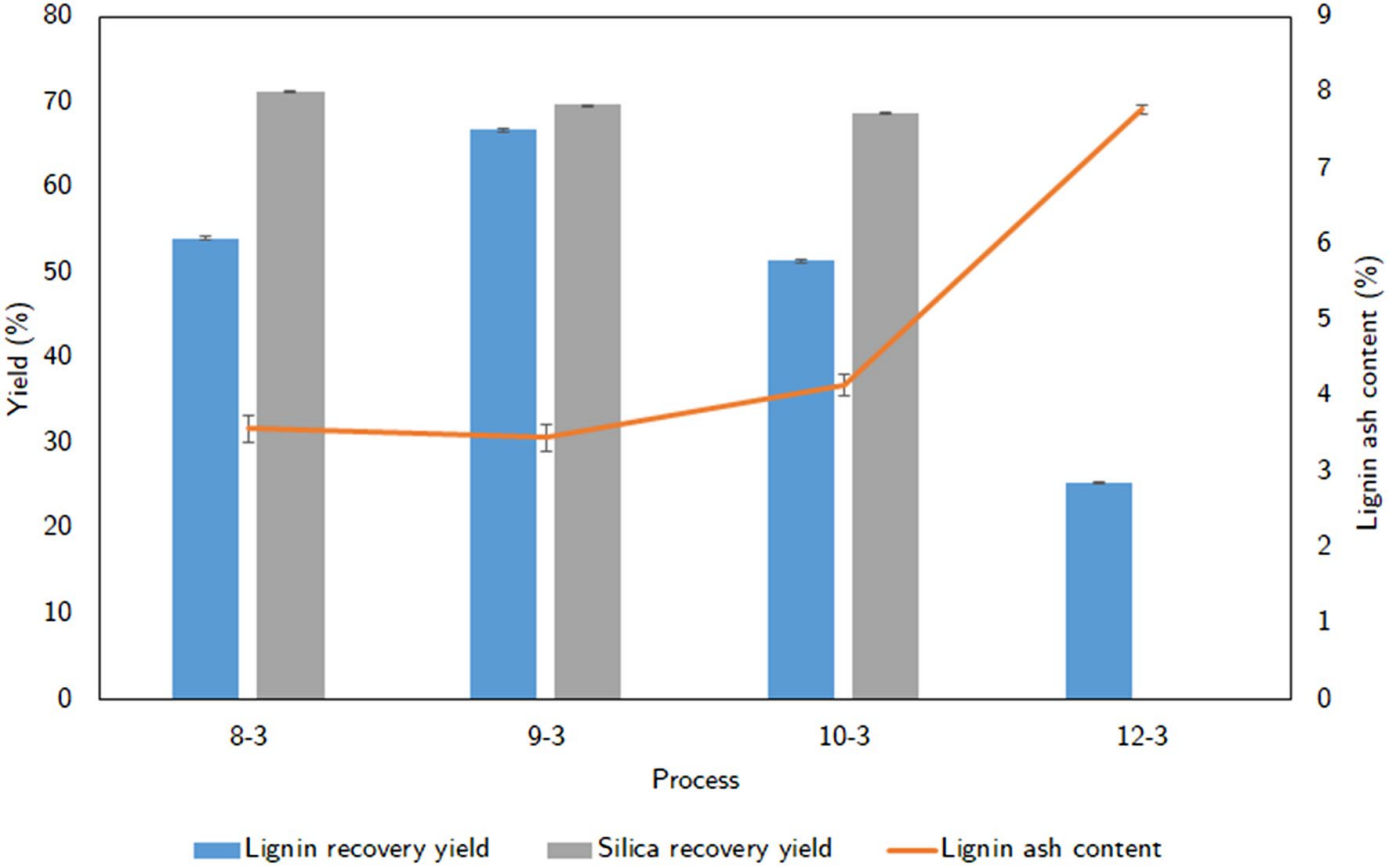
The recovery yield of lignin and silica, the ash content of lignin in difference processes.

In order to determine the optimal pH for removing silica out of black liquor and recovering desilication lignin, the black liquor was adjusted to pH 8, 9, 10, and then to pH 3 to find out the appropriated pH value. The results were shown in Fig. 5 indicating the amount of silica obtained at pH 8 is the highest followed by pH 9 and 10. While the yield of lignin at the 9–3 process is higher than the 8–3 process and the purity of two processes is not significantly different. Therefore, when considering the economy, the 9–3 process is outstanding for desilication lignin production and recovery of silica.

### The two-step acidification for recovery lignin with high purity

**Figure 6:**
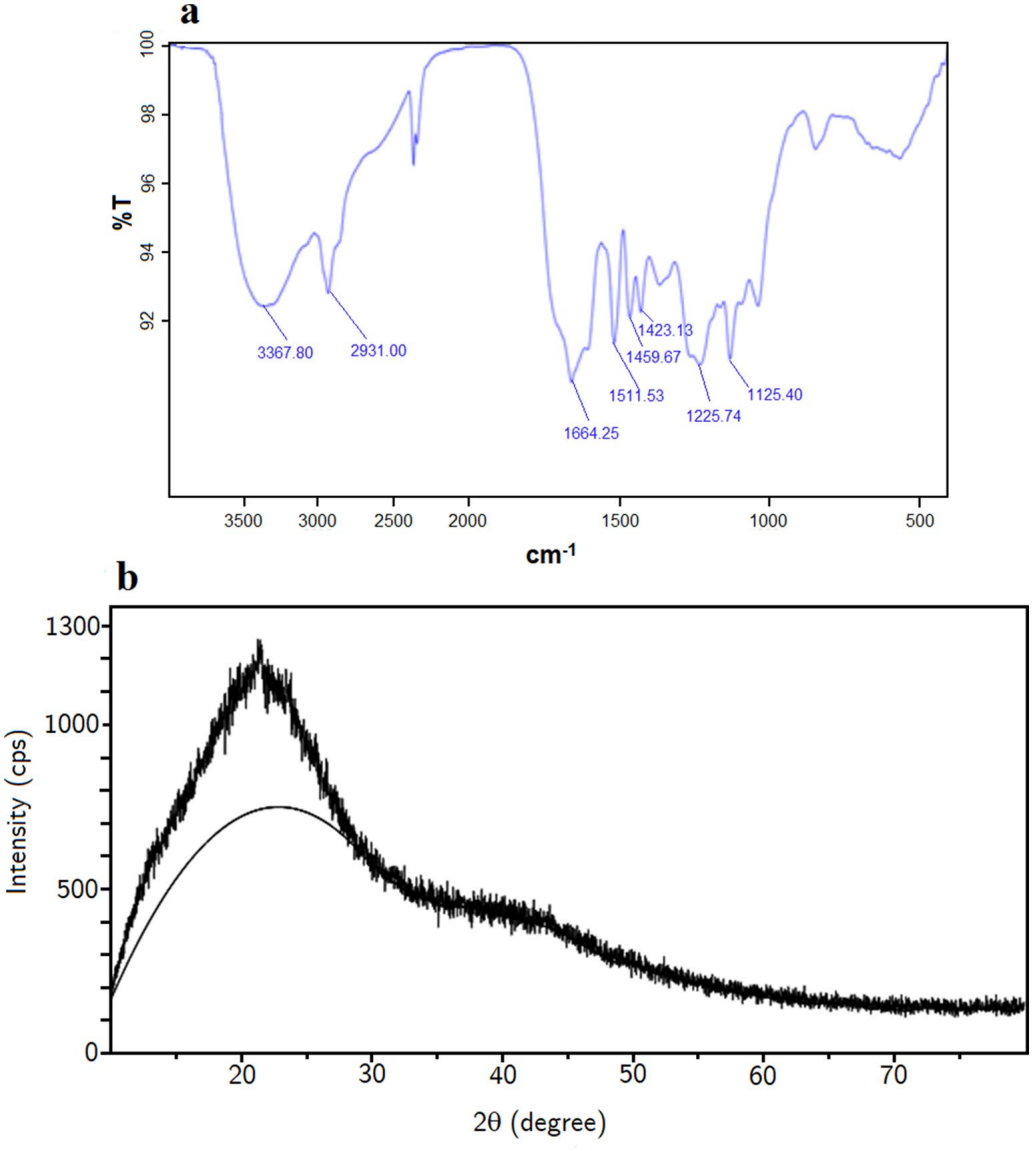
FTIR and XRD spectrums of lignin in a two-steps process.

**Figure 7:**
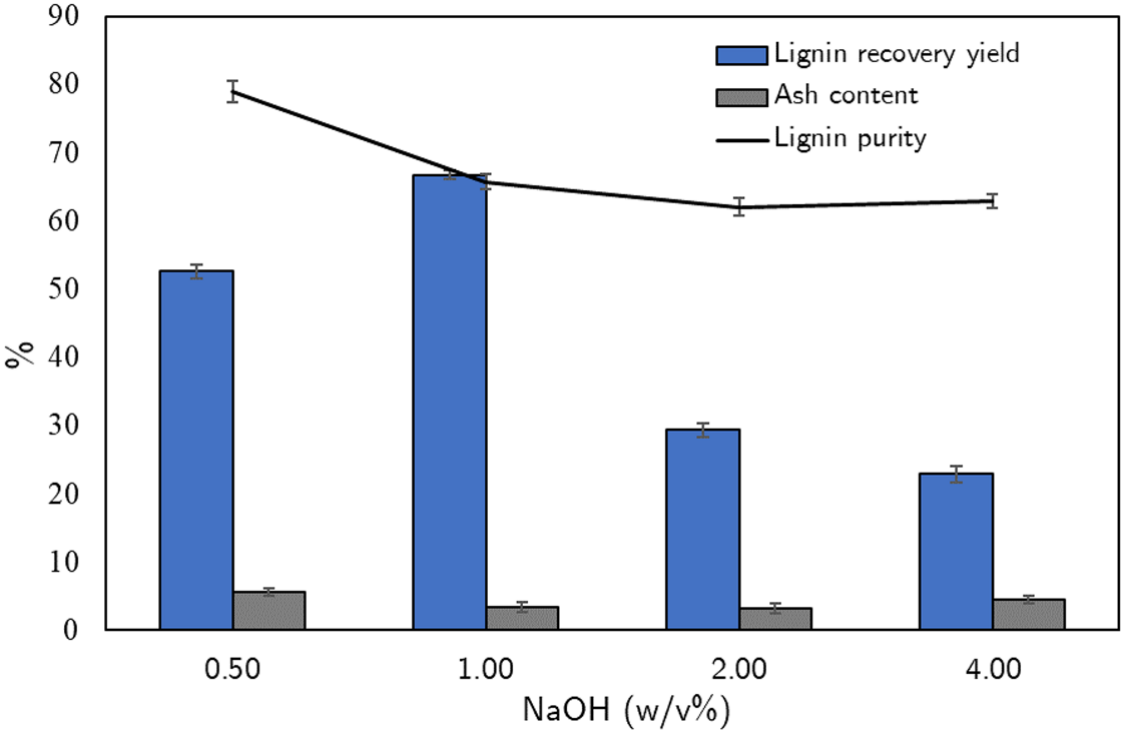
The recovery yield, the purity, and the silica content of lignin in several concentrations of NaOH.

The target of this process is to obtain pure lignin by removing silica-based components out of the black liquor before recovering lignin. According to the results of all experiments, that process is divided into 2 steps. First-step, black liquor was acidified to pH 9 to remove silica and the next step is lignin recovery at pH 3. The lignin precipitate from this process will be analyzed to assess the lignin component, the analytical results are shown in Fig. 6. The FTIR spectrum of the precipitate from the two-step process (Fig. 6a) demonstrates that the presence of a special band of lignin is around 1510 cm^−1^ and 1605 cm^−1^ for aromatic skeletal vibration (C=C) of lignin (guaiacyl or syringyl). The effect of the silica band is insignificant because of the absence of silica bands around 458–561 cm^−1^. However, the XRD analysis results (Fig. 6b) show that there are 2 peaks around 22°. This proves that the extracted lignin is not completely removed from the silica, although the ash content is 3.46% of total precipitation weight equivalent to 81.11% silica reduction (Fig. 7). Therefore, the two-steps process could be presumed as a method to obtain pure lignin.

### Effect of sodium hydroxide concentration on lignin yield and recovery in the 2-step process towards industrial implementation

However, in order to apply the proposed 2-step lignin and silica recovery process at the industrial scale, it must satisfy in many factors such as economy, efficiency, and safety during implementation. In particular, the concentration of NaOH is one of the important factors. The higher the NaOH concentration is used, the higher the safety risks that take place in the mixing or operation process. Moreover, the operating equipment should have special requirements for high corrosion resistance. Therefore, the experiment is conducted to find the appropriate NaOH concentration for the recovery of lignin from the black liquor by a 2-step process. The effect of NaOH concentration on the lignin recovery yield and the purity of lignin is shown in Fig. 7. The result depicts that NaOH 1w/v% process had the most potential for recovery lignin with 66.75% lignin recovery yield followed by NaOH 0.5 w/v% process with 52.64% lignin recovery yield, while the silica content of NaOH 1 w/v% and NaOH 2 w/v% process is 3.46% and 3.22% equivalent to 94.38% and 99.98% of removed silica content, respectively. The obtained lignin from the process using NaOH 0.5 w/v% has the highest purity of 78.91%. The reason is that at high alkaline concentration, the polysaccharides in rice straw were degraded and dissolved which leads to decreasing lignin recovery yields ^50^. Therefore, the study suggests the process using NaOH 1 w/v% in the pretreatment process and following the two-step process as shown in Supplemental Fig. S1 because of the silica reduction, lignin recovery yield, and purity.

## Conclusions

The effects of pH value on the behavior of the precipitates of lignin and silica from the black liquor of rice straw have been comprehensively investigated by step-by-step acidification with dilute H_2_SO_4_. The color of the precipitate changes from golden brown to dark brown with decreasing pH value, whereas the opposite trend is true for the filtrate. The pH value of 3 is demonstrated to recover lignin most effectively based on chemical structure analysis, while a pH range of 10–8 was demonstrated to remove silica from the black liquor. A two-step acidification process was developed with prior acidification in diluted sulfuric acid to pH 9 and removal of silica, followed by acidification to pH 3 for optimal lignin recovery. These findings are valuable in mass production to identify the lignin recovery in acidification of the black liquor. Moreover, in the view of industrialization, with utilizing low concentration basic chemical as NaOH 1% and H_2_SO_4_ 20%, this method was not only producing value-add products but also saving energy and lowering the waste to the environment because of its simplicity and efficiency. The precipitate obtained at pH 3 shows the highest lignin purity of 65.74%, the recovery yield of 66.75%, and the silica reduction of 94.38% by using diluted sodium hydroxide with a low concentration of 1w/v%.

## Supplementary information

Supplementary Information.
