# Author Correction: The novel method to reduce the silica content in lignin recovered from black liquor originating from rice straw

**DOI:** 10.1038/s41598-021-97180-z

**Published:** 2021-09-07

**Authors:** Nghi H. Do, Hieu H. Pham, Tan M. Le, Jeroen Lauwaert, Ludo Diels, An Verberckmoes, Nga H. N. Do, Viet T. Tran, Phung K. Le

**Affiliations:** 1 Institute of Natural Products Chemistry – Vietnam Academy of Science and Technology, 18 Hoang Quoc Viet, Hanoi, Vietnam; 2 Refinery and Petrochemicals Technology Research Center (RPTC), Ho Chi Minh City University of Technology (HCMUT), 268 Ly Thuong Kiet Street, Ho Chi Minh City, Vietnam; 3 Vietnam National University Ho Chi Minh City (VNU-HCM), Linh Trung Ward, Thu Duc District, Ho Chi Minh City, Vietnam; 4 Industrial Catalysis and Adsorption Technology (INCAT), Department of Materials, Textiles and Chemical Engineering (MaTCh), Faculty of Engineering and Architecture, Ghent University, Valentin Vaerwyckweg 1, 9000 Ghent, Belgium; 5 Institute of Environment and Sustainable Development (IMDO), University Antwerp, Groenenborgerlaan 171, 2020 Antwerp, Belgium; 6 Flemish Institute for Technological Research (VITO), Boeretang 200, 2400 Mol, Belgium

Correction to: *Scientific Reports* 10.1038/s41598-020-77867-5, published online 04 December 2020

In the original version of the Article, Figure 7 was a duplication of Figure 5.

The original Article has been corrected.

