# Supplementary material for: The novel method to reduce the silica content in lignin recovered from black liquor originating from rice straw
Source: Sci Rep. 2020 Dec 4;10:21263. doi: 10.1038/s41598-020-77867-5 (PMC7718241; doi:10.1038/s41598-020-77867-5)
Supplement: Supplementary file 1 — Supplementary Information. [file 41598_2020_77867_MOESM1_ESM.pdf]

Supplementary information

The novel method to reduce the silica content in lignin recovered from black liquor originating from rice straw

Nghi H. Do[a], Hieu H. Pham[b,c], Tan M. Le[b,c], Jeroen Lauwaert[d], Ludo Diels[e], An Verberckmoes[d], Nga H.N. Do[b,c], Viet T. Tran[b,c], Phung K. Le[b,c]*.

[a]Institute of Natural Products Chemistry – Vietnam Academy of Science and Technology; 18 Hoang Quoc Viet, Hanoi, Vietnam)

[b]Refinery and Petrochemicals Technology Research Center (RPTC), Ho Chi Minh City University of Technology (HCMUT), 268 Ly Thuong Kiet Street, Ho Chi Minh City, Vietnam

[c]Vietnam National University Ho Chi Minh City (VNU-HCM), Linh Trung ward, Thu Duc District, Ho Chi Minh City, Viet Nam

[d]Industrial Catalysis and Adsorption Technology (INCAT), Department of Materials, Textiles and Chemical Engineering (MaTCh), Faculty of Engineering and Architecture, Ghent University, Valentin Vaerwyckweg 1, 9000 Ghent, Belgium.

[e]Institute of Environment and Sustainable Development (IMDO), University Antwerp, Groenenborgerlaan 171, 2020 Antwerp, Belgium.

[f]Flemish Institute for Technological Research (VITO), Boeretang 200, 2400 Mol, Belgium.

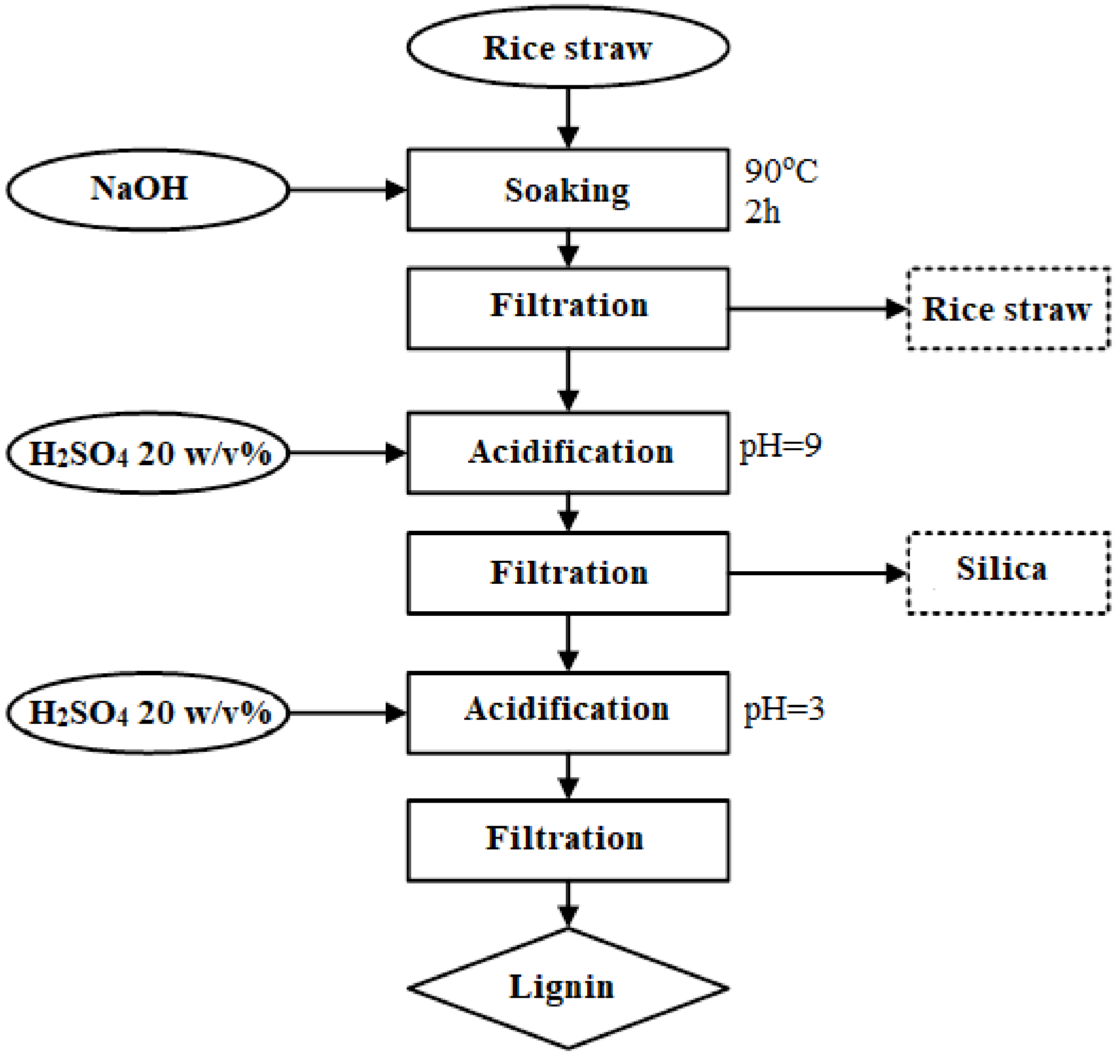

Figure S1. Schematic representation of the process of recovery of lignin and silica from rice straw.

The lignin and silica recovery process were including 3 steps: first is the pretreatment step, the rice straw was soaked with NaOH 1w/v% in 2h at 90°C to gain the black liquor. The liquor was continuously treated with H_2SO_4 20w/v% in next 2 steps. At the second step, the black liquor was adjusted from pH 12.4 to pH 9 and sedimented in 36h for silica gelation. The silica gelation can be filtrated by vacuum filter, clothes filter or press filter. Lignin was recovered in the final step by adjusting to pH 3 and waiting at least 12h for lignin precipitation. The lignin precipitation was separated by vacuum filter, clothes filter or press filter.

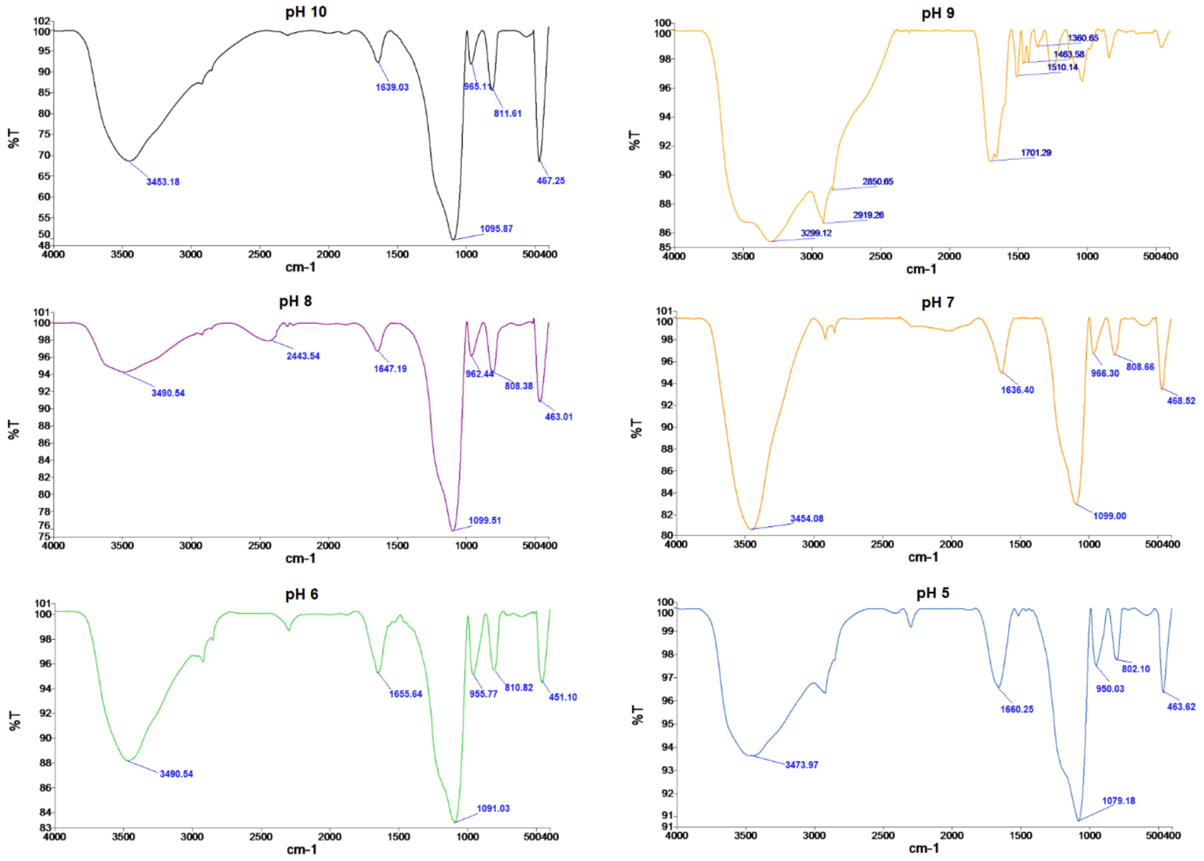

Figure S2: FTIR spectrum of the precipitate from pH 10 to pH 5

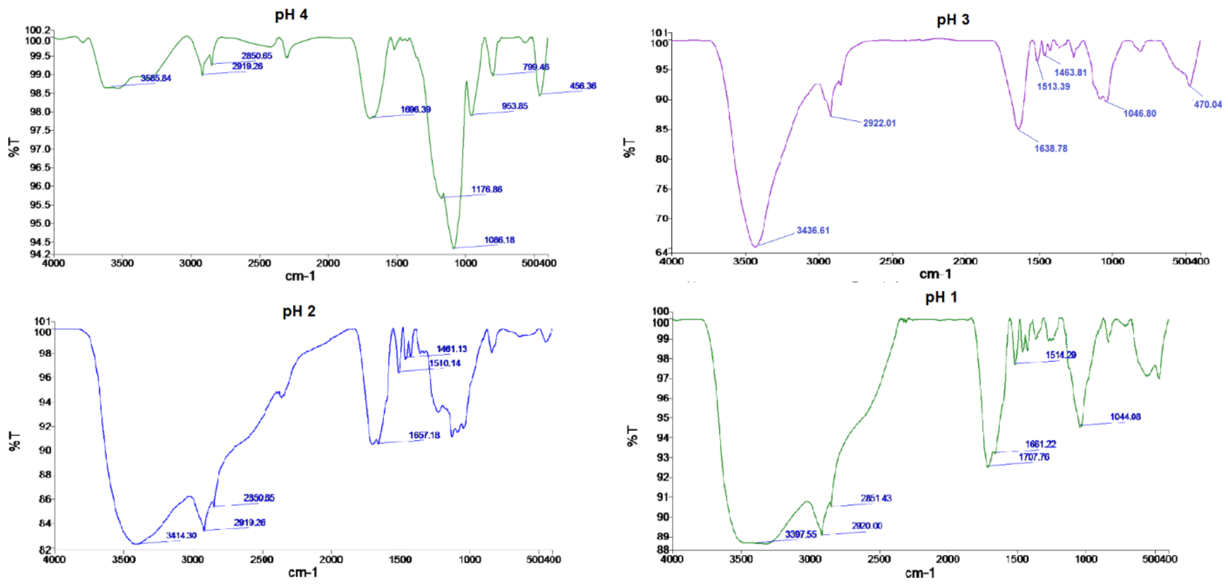

Figure S3: FTIR spectrum of the precipitate from pH 4 to pH 1

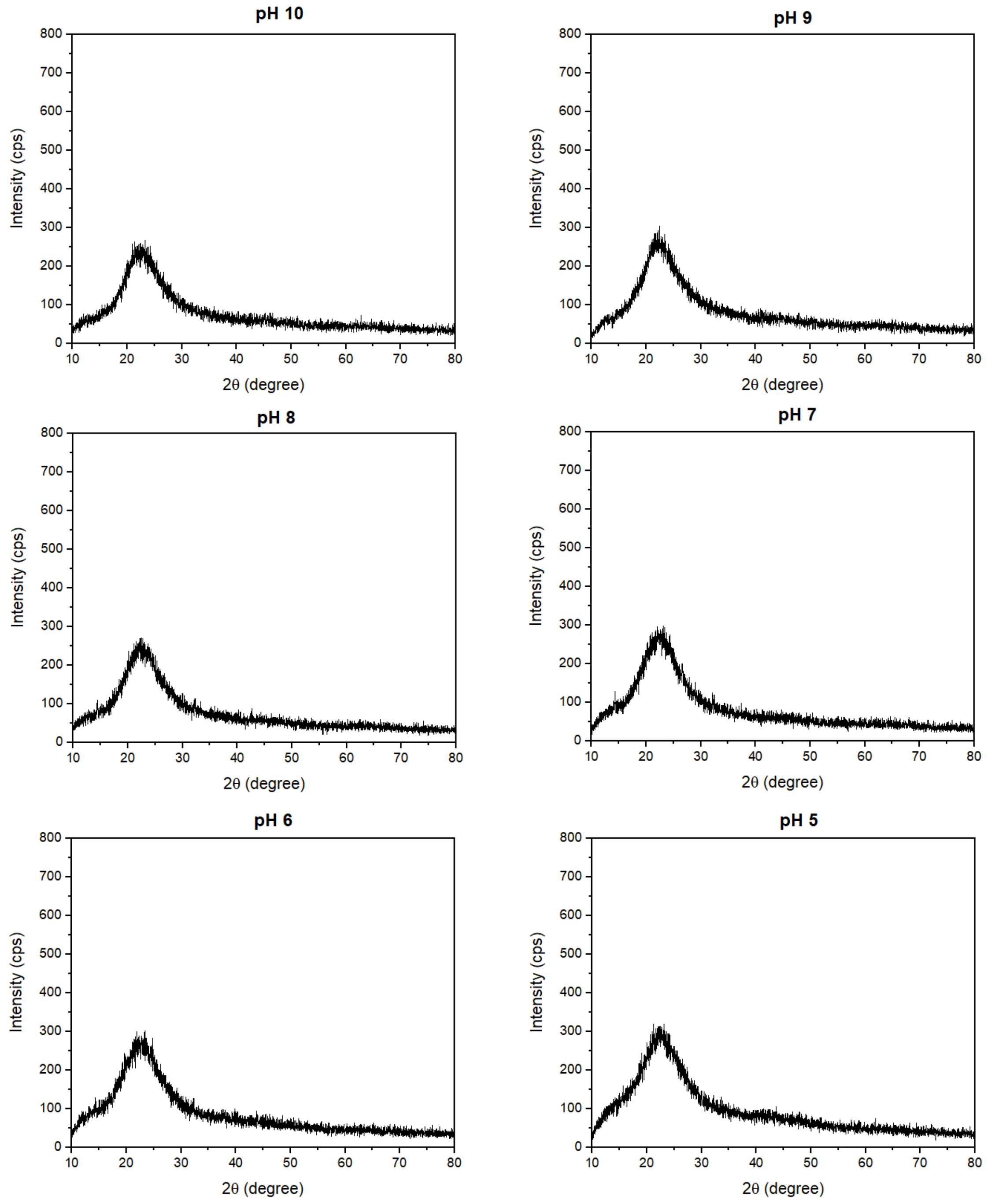

Figure S4: XRD spectrum of the precipitate from pH 10 to pH 5

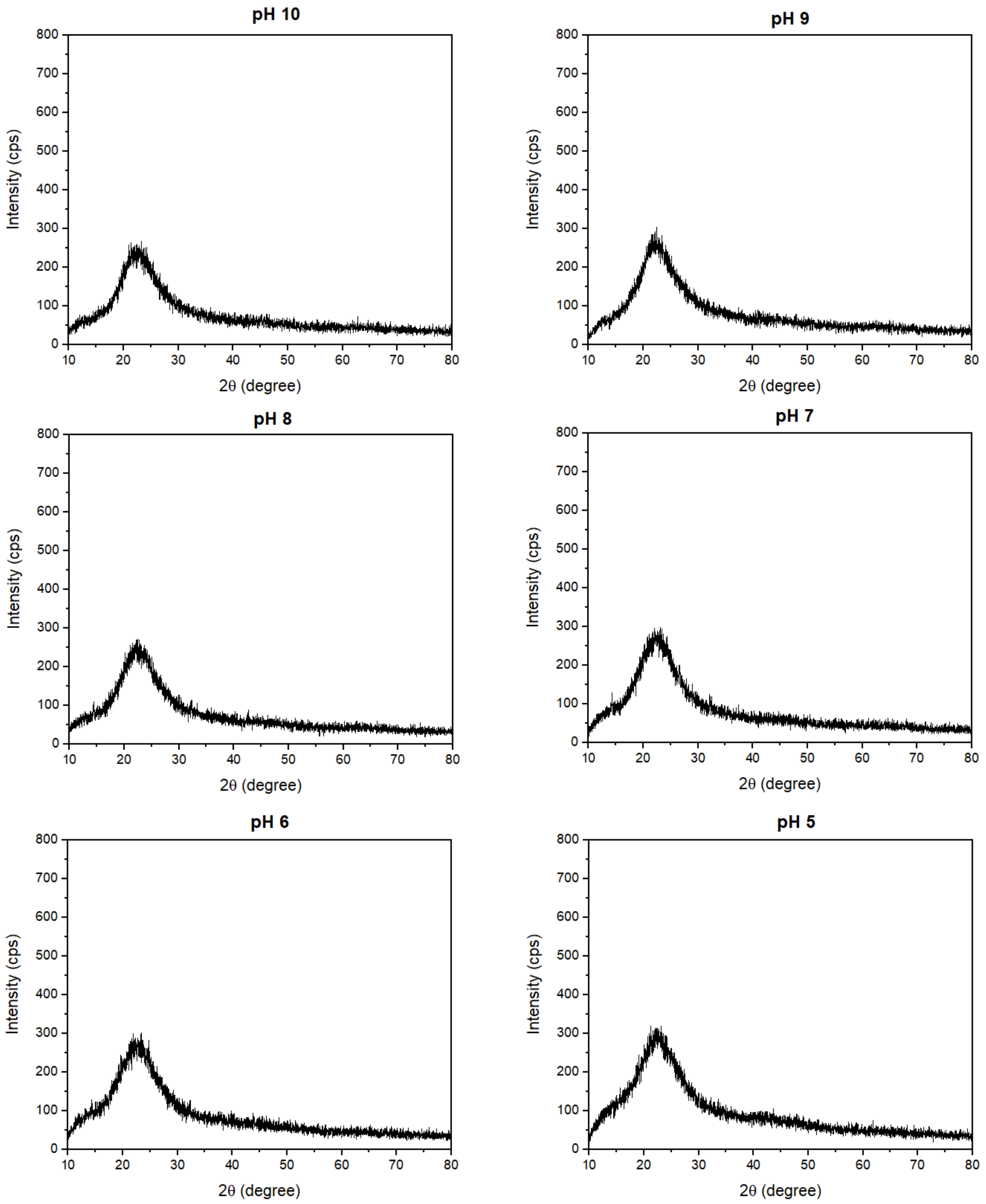

Figure S5: FTIR spectrum of the precipitate from pH 4 to pH 1

Table S1. The composition of precipitation ash in difference pH (pH 10,9,8), determined by XRF

Element	Content (%)		
	pH 10	pH 9	pH 8
SiO_2	82,6	87,5	97,2
ZrO_2	12,6	8,88	1,4
CaO	1,68	1,21	0,457
Others	3,12	2,41	0,943

Table S2 FTIR frequency range and functional groups present in the sample.

Frequency range (cm^{-1})	Functional group
3500-3100	-OH stretching
2800-3750	asymmetric stretching and bending vibrations of silanol OH groups (SiO–H)
2900-2935	-CH stretching
1604-1735	C=O stretching of carbonyl, carboxyl and acetyl group and of xylans
1510-1605	Aromatic skeletal vibration (C=C) of lignin
1455-1465	-CH_3O stretching vibration
1046-1099	-CO stretching vibration
950-1000	Si-O-Si asymmetric stretching
800-833	-CH bonds in associated to aromatic rings.
458-561	Si-O-Si bending
